# Toxicity Responses from Tributyltin Chloride on Haarder (*Planiliza haematocheila*) Livers: Oxidative Stress, Energy Metabolism Dysfunction, and Apoptosis

**DOI:** 10.3390/cimb47070526

**Published:** 2025-07-08

**Authors:** Changsheng Zhao, Anning Suo, Dewen Ding, Wencheng Song

**Affiliations:** 1 Institute for the Control of Agrochemicals, Ministry of Agriculture and Rural Affairs, Beijing 100125, China; zhaochangsheng@agri.gov.cn (C.Z.);; 2 Key Laboratory of Pesticide Assessment, Ministry of Agriculture and Rural Affairs, Beijing 100125, China

**Keywords:** tributyltin chloride, oxidative stress, energy metabolism disorder, apoptosis

## Abstract

In coastal waters, tributyltin chloride (TBTC), a persistent organic pollutant, is extensively present. It is uncertain, therefore, if exposure to TBTC can harm haarders and how. This study exposed the fish for 60 days in order to investigate the molecular mechanism of haarder following TBTC poisoning. Our findings demonstrated that growth indices dropped, liver tissue was damaged, and the liver’s total tin concentration rose following TBTC exposure. Furthermore, we discovered that blood reactive oxygen species rose while total blood cell count decreased. As malondialdehyde levels rose, total antioxidant capacity and antioxidant enzyme activity (superoxide dismutase, catalase, and glutathione peroxidase) were markedly reduced. After being exposed to TBTC, liver cells displayed clear signs of apoptosis. Differentially expressed genes were primarily linked to oxidative stress, energy metabolism, and apoptosis, according to the transcriptome study of livers. Overall, the long-term stress of TBTC resulted in the antioxidant system being harmed, as well as serious malfunction of the energy metabolism and apoptotic response.

## 1. Introduction

Since the 1950s, tributyltin (TBT), one of the most hazardous organic pollutants, has been utilized as an antifouling coating for fishing nets and ships as well as an industrial and agricultural pesticide [1]. The continuous release of TBT has led to its highly concentrated presence in coastal waters, especially near ports and docks [2,3]. TBT is thought to be extremely persistent because its half-life in sediments can last for several decades [4]. Since TBT is a frequent plastic stabilizer and catalyst, it has become an additional source of TBT pollution as the issue of plastic pollution has grown more widespread and is now a global concern [5]. Despite a 2008 large-scale ban on TBT as an antifouling paint for ships, its longevity and several extensive sources have resulted in its ubiquitous presence in sediments, water bodies, and even creatures, endangering the aquatic ecosystem and its inhabitants [6,7]. According to international investigations, TBT levels are as high as 111.29 μg Sn/L in water [8] and 10 μg Sn/g in marine sediments [9], respectively. These levels are far higher than the acceptable threshold (1 ng/kg to 50 ng/g organo-tin in seawater) and would undoubtedly result in extremely high ecological concerns [10].

Reactive oxygen species (ROS)-mediated oxidative stress is defined as the disruption of an organism’s dynamic balance [11]. Pollutant-induced ROS production and oxidative damage to key oxidative stress molecules are common toxicological mechanisms in teleosts that contribute to their pathogenicity [12]. According to Burgos-Aceves et al. [13], bony fish have developed a comparatively well-developed defense system that allows them to adjust to oxidative stress brought on by contaminants. Fish include a number of antioxidant enzyme systems, including superoxide dismutase (SOD), catalase (CAT), and glutathione peroxidase (GPx), that deal with reactive oxygen-induced damage and reflect the body’s stress resistance capabilities [14]. Furthermore, lipid peroxidation is a reaction process exhibited by fish when exposed to contaminants, as indicated by the presence of malondialdehyde (MDA) [15]. According to studies on the damage to the antioxidant defense system in abalone cells or the worsening of lipid peroxidation, TBT induces oxidative stress [16]. Moreover, ATPases (including Na^+^-K^+^-ATPase and Ca^2+^-Mg^2+^-ATPase) are a class of enzymes that regulate energy metabolism. Exogenous stress factors interfere with the metabolic pathways of energy generation in teleost fish, inhibiting the activity of these enzymes [17,18]. Evidence shows that TBT influences mitochondrial Mg ATPase activity in aquatic animals such as Mediterranean mussels [19]. Studies suggest that pollutants in the ocean additionally influence the tissue morphology of various aquatic animals (including bony fish) and induce their apoptosis [20,21,22]. Numerous studies have found that apoptosis is directly linked to the generation of excessive ROS [23,24,25]. As a coastal and demersal bony fish, the habitat of haarder (*Planiliza haematocheila*) may overlap with places having high TBT residues [26]. Haarder’s habit of feeding on debris in the sediment increases the risk of TBT poisoning [27].

The chronic (60-day) toxic reaction of haarder to TBT (represented by tributyltin chloride, or TBTC) was identified in this investigation. Prior to testing the growth index, antioxidant system response, and blood oxidative stress response indicators, the livers’ total tin content was determined. The effects of various TBTC dosages were then investigated by looking at the livers’ histological sections and apoptosis. Several sets of functional liver genes were thoroughly examined in order to learn more about the transcriptome-level mechanism of action of TBTC on haarder.

## 2. Materials and Methods

### 2.1. Animals and Chemicals

A farm in Zhuhai, Guangdong Province, China, provided *Planiliza haematocheila* (6 months old), with an average length of 13.5 ± 1.12 cm and an average weight of 24.2 ± 4.57 g. A total of 36 haarder individuals were initially reared domestically in 150 L glass tanks for two weeks, receiving the proper commercial feed twice a day to meet the needs of the experiment (at 8:00 a.m. and 16:00 p.m.). The remaining feed and excrement in the tank water were promptly cleaned up, and it was maintained at a temperature of 25.6 ± 0.5 °C and a salinity of 3.2 ± 0.6‰. The Chinese Academy of Sciences’ Animal Research and Ethics Committee gave its approval for our work (SCSIO-IACUC-2021-000156), and we followed all ethical and animal welfare guidelines.

### 2.2. Tributyltin Chloride (TBTC) Exposure Experiments

Treatment groups L, M, and H were established based on the preliminary experiment results of haarder exposed to TBTC [28]. The dose groups were 3.4 (L group), 34.4 (M group), and 344.2 (H group) ng/L, respectively. TBTC was insoluble in water; hence, dimethyl sulfoxide (DMSO) was used as a co-solvent. A V_DMSO_:V_water_ ratio of 1:10^7^ was used to prepare the experimental water for the control group (C group) and the three treated groups (L, M, and H). For this TBTC acute exposure experiment, 36 fish were chosen at random and split up into a total of 12 tanks (3 replicate tanks per group, with 3 fish in each replication). Every day, a third of the tank water was added to guarantee the stability of each TBTC exposure concentration. At 60 days, three fish were randomly taken out of each tank and put to death on ice using 100 mg/L MS-222. A random sample was taken from each group’s surviving fish (n = 9). The fish’s livers were sampled for use in subsequent studies when their length and weight were determined.

### 2.3. Total Tin Content in Haarder

Total tin (Sn) in livers was detected according to modified methods described by the State Standard of the People’s Republic of China [29]. A suitable quantity of each homogenized liver sample was digested with 5% HNO_3_ overnight in a microwave digestion system, followed by a 30 min heating period at 100 °C in an ultrasonic water bath. The samples were diluted and equally mixed before being analyzed using inductively coupled plasma–mass spectrometry (Agilent 7900, Santa Clara, CA, USA). For total tin, its limit of detection was 1.1 ng/g on a wet weight basis.

### 2.4. Growth Indices’ Measurement

Two morphological indices, hepatosomatic index (HSI) and Fulton’s condition factor (FCF), based on the weight and length of haarder respectively, were calculated according to Maddock and Burton [30]. The calculation was performed as follows: HSI = 100W_L_/W_T_, where W_L_ is the liver weight (in g), and W_T_ is the total weight (in g); and FCF = 100W_T_/L_T_^3^, where L_T_ is the total length (in cm).

Frozen liver samples for the analysis of ATPase activity (Na^+^-K^+^-ATPase and Ca^2+^-Mg^2+^-ATPase) were homogenized on ice with 1 × phosphate-buffered saline (pH 7.2). At 37 °C, 100 μL of centrifuged supernatant and the appropriate quantity of mixed reagents were subjected to an enzymatic reaction. After standing at room temperature for 5 min, the absorbance was measured using a spectrophotometer (Shimadzu, Kyoto, Japan) at a wavelength of 636 nm.

### 2.5. Antioxidant Capacity in Livers

Frozen liver samples were used to measure the activities of SOD, CAT, and GPx, the content of MDA, and the total antioxidant capacity (T-AOC). Using appropriate assay kits (Nanjing Jiancheng Bioengineering Institute, Nanjing, China), all of the aforementioned biochemical indicators were measured. One activity unit of SOD was defined as the amount of SOD corresponding to 50% inhibition rate of SOD in each mL of reaction solution [31]. The suspension of homogenized liver samples was mixed into the reaction reagents, and the absorbance was measured at a wavelength of 550 nm. For CAT, its one activity unit was the amount of decomposing one micromole of H_2_O_2_ per second per milliliter of reaction solution [32]. An amount of 200 μL of the mixed reaction solution was taken out and placed in a 96-well plate, and the absorbance of each well was measured with a microplate reader (Thermo Scientific, Waltham, MA, USA) at 405 nm. One activity unit of GPx was that every 0.1 mg protein reacts at 37 °C for 5 min, and the concentration of glutathione in the reaction system decreased by 1 μmol/L after deducting the non-enzymatic reaction [33]. After the enzymatic reaction and chromogenic reaction, the reaction solution was evenly mixed and stood for 15 min, and its absorbance was measured by a spectrophotometer (Shimadzu, Kyoto, Japan) at the wavelength of 412 nm. The ground liver tissue was diluted into a 10% homogenate, and the supernatant was taken after centrifugation to prepare for the later determination of MDA content. After the supernatant was bathed in boiling water for 40 min, it was centrifuged at 3500 rpm for 10 min, and the absorbance of the supernatant was measured at 532 nm [34]. T-AOC was determined by the ferric reducing ability of plasma method [35]. The reaction solution was reacted at room temperature for 6 min, and the absorbance was measured at the wavelength of 593 nm. The Bradford method was employed to measure the protein in the liver, with bovine serum albumin serving as the reference [36].

### 2.6. Total Blood Cell Count and Intracellular Reactive Oxygen Species (ROS) Analyses

A 1-mL syringe with an equal volume of pre-cooling anticoagulant solution was used to draw 200 μL of blood from each fish. The total blood cell count value was recorded when the diluted blood sample was placed on a hemocytometer and examined using an optical microscope (Olympus, Tokyo, Japan).

The level of intracellular ROS was usually expressed by the level of respiratory burst (RB) [37]. A probe called 2′,7′-dichloro fluorescein diacetate (DCF-DA) was employed in the detection. An amount of 200 μL of the blood samples (1 × 10^6^ cells/mL) was incubated in triplicate with DCF-DA (20 μM) for 30 min in the dark at room temperature. A FACSCalibur Flow Cytometer (BD Biosciences, San Jose, CA, USA) was used to assess the fluorescence of each sample. RB levels were calculated by averaging the fluorescence of DCF values.

### 2.7. Histological and Apoptosis Detection

The liver tissues that were taken were fixed for 24 h using a 4% paraformaldehyde solution. After being dried in ethanol, embedded in paraffin, and sectioned into slices, the sections were cleaned, dewaxed, and stained for eight minutes with cold hematoxylin and three minutes with eosin. A light microscope (Leica, Wetzlar, Germany) was used to view each liver section.

To detect liver cell apoptosis, we employed the terminal-deoxynucleotidyl transferase (TdT)-mediated dUTP nick end labeling (TUNEL) method. The fixed liver slices were deparaffinized and rehydrated, and then they were incubated with proteinase K at 25 °C for 20 min. Each section was stained with 4′,6-diamidino-2-phenylindole and incubated with TdT. All sections were then examined under a fluorescence microscope (Zeiss, Oberkochen, Germany) with a 40× lens magnification. Three samples from each treatment or control group were selected for this investigation. Green fluorescein labeling was used to identify the apoptotic nuclei after all liver cells were stained blue. The ImageJ program (1.53k, National Institutes of Health, Bethesda, MD, USA; Java 1.8.0_172) was used to estimate the apoptotic indices [38].

### 2.8. RNA-Seq and Bioinformatics Analysis

After TBTC exposure, the livers of haarder fish in the H group and the C group were collected for their transcriptome sequencing. The TRIzol Reagent Kit (Invitrogen, Carlsbad, CA, USA) was used to extract total RNA from three liver samples according to the manufacturer’s protocol. The end-repaired, poly (A)-added, and ligated pure cDNA fragments were linked to Illumina sequencing adapters (Illumina, San Diego, CA, USA). The library was created by size-selecting the ligation products using agarose gel electrophoresis, PCR amplification, and an Illumina NovaSeq 6000 system. The raw reads were filtered using the fastp program (v0.18.0) [39] to remove adaptor reads and any low-quality reads, leaving only a high-quality clean data set. After that, unigenes were created by de novo assembly using the Trinity short reads assembling tool [40]. The quality of the resulting assembly was evaluated using N50 values, Q20, Q30, sequence length, and Benchmarking Universal Single-Copy Orthologs (BUSCO) (http://busco.ezlab.org/).

Fragments Per Kilobase of transcript per Million mapped reads (FPKM) was used to compute and standardize the unigene expression and abundance values [41]. Based on the expression information of each sample, principal component analysis (PCA) was used to study the distance relationship between samples through the idea of dimension reduction. The annotation of unigenes included their protein functional annotation, pathway annotation, COG/KOG functional annotation, and Gene Ontology (GO) annotation. The analysis of differentially expressed genes (DEGs) between two separate groups was conducted using the DESeq2 package for R (version 4.1.0) [42]. DEGs were defined as genes with a false discovery rate (FDR) < 0.05 and |log2FC| > 1. To acquire GO function categorization annotations for the genes and identify those that are considerably enriched, the GO enrichment analysis was conducted. The most important biochemical and signal transduction pathways were identified by Kyoto Encyclopedia of Genes and Genomes (KEGG) pathway analysis for significance enrichment. Raw sequencing data was deposited with GenBank in a Sequence Read Archive under the BioProject number PRJNA869536.

### 2.9. Quantitative Real-Time PCR (qRT-PCR) Detection of Differentially Expressed Genes (DEGs)

Ten DEGs were selected for the qRT-PCR analysis to verify the accuracy of the transcriptome data. The cDNA samples were extracted from the livers. Their specific primers were designed using Primer Premier software (version 5.00). The elongation factor 1 alpha (*EF1-α*) gene from haarder *P. haematocheila* (GeneBank accession number: AXB71857) was quantified and served as an internal control to normalize the qRT-PCR results. Prior to conducting this qRT-PCR experiment, the specificity and efficiency of the chosen primers were determined using PCR and electrophoresis on a 1.2% agarose gel, respectively. Every reaction was carried out in a 20 μL total volume consisting of 10 μL of 2 × SYBR Premix Ex Taq™ II (TaKaRa, Kyoto, Japan), 2 μL of the cDNA template (50 ng total RNA), 0.4 μL of ROX Reference Dye (50×), 0.4 μL of both sense and anti-sense primers (10 μM), and 6.8 μL of PCR-specific ddH_2_O. The PCR program consisted of 1 cycle of 95 °C for 3 min, followed by 38 cycles of 96 °C for 15 s and 59 °C for 30 s. qRT-PCR was performed using the 2^−△△Ct^ method in ABI 7500 software (version 2.3, Applied Biosystems, San Jose, CA, USA) [43].

### 2.10. Statistical Analysis

GraphPad Prism v9.0.0 software (San Diego, CA, USA, www.graphpad.com) is used to statistically analyze the data. The mean ± standard error of mean (SEM) is how the findings are displayed. To find significant differences, the sample means are compared using Dunnett’s test and one-way analysis of variance (ANOVA). *, **, and *** stand for the thresholds at *p* < 0.05, *p* < 0.01, and *p* < 0.001, respectively.

## 3. Results

### 3.1. Total Tin Contents in Livers

**Figure 1:**
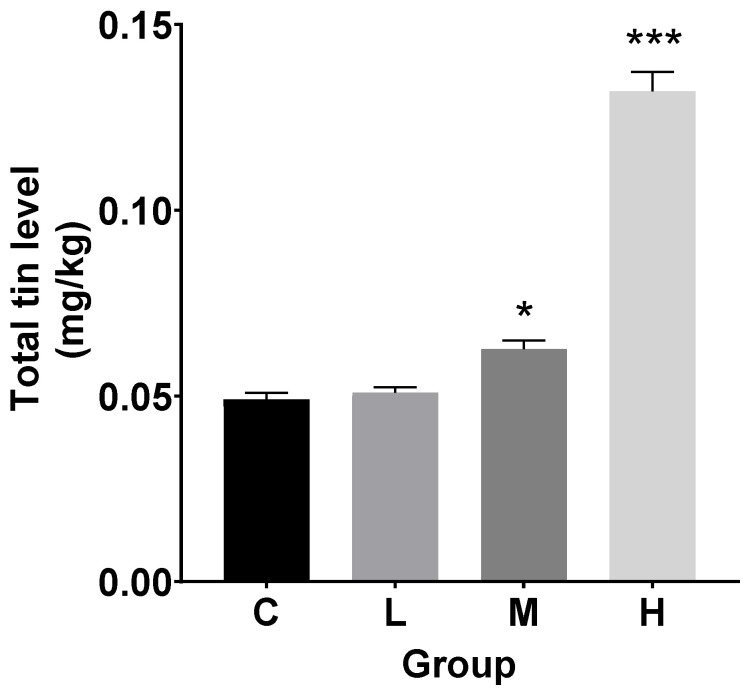
Total tin content in the livers after 60 d of TBTC exposure. The livers were obtained from three individuals in the treatment and control groups (n = 3). A statistically significant difference from the control is shown by an asterisk (* at *p* < 0.05 and *** at *p* < 0.001).

After 60 days of chronic TBTC exposure, the liver’s total tin content was measured. As seen in Figure 1 and Figure S1, the M and H treatment groups’ total tin levels were clearly higher than the control group’s. The level of total tin was observed to be dose-dependent as the TBTC dose increased in the treatment groups. Specifically, compared to the control group, the M and H groups’ liver total tin levels increased by 0.3 and 1.7 times, respectively.

### 3.2. Hepatosomatic Index (HSI), Fulton’s Condition Factor (FCF), and Histological Index

**Figure 2:**
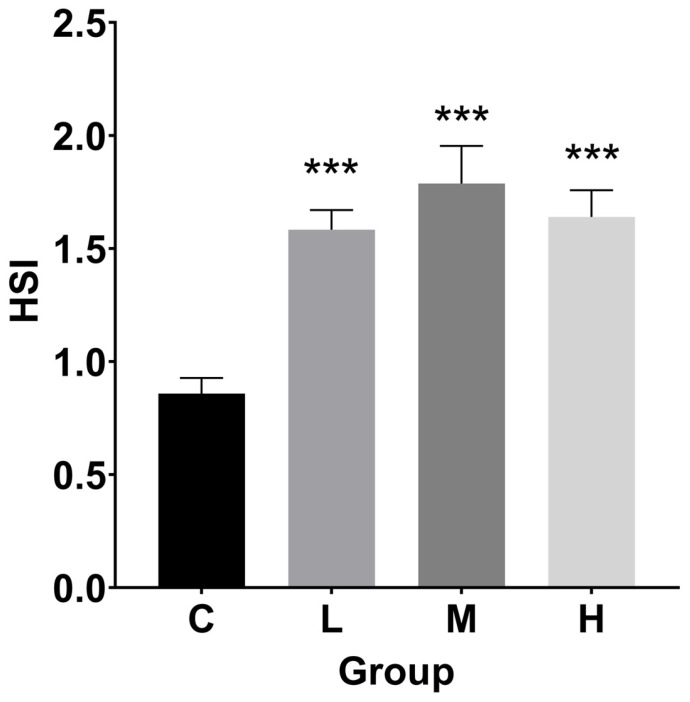
Hepatosomatic index (HSI) for TBTC-treated haarder fish (n = 9). A statistically significant difference from the control is shown by an asterisk (*** at *p* < 0.001).

As seen in Figure 2, the HSI values rose following a 60-day exposure. The HSI in the treated groups increased 0.8, 1.1, and 0.9 times, respectively, in comparison to the control group (*p* < 0.001).

**Figure 3:**
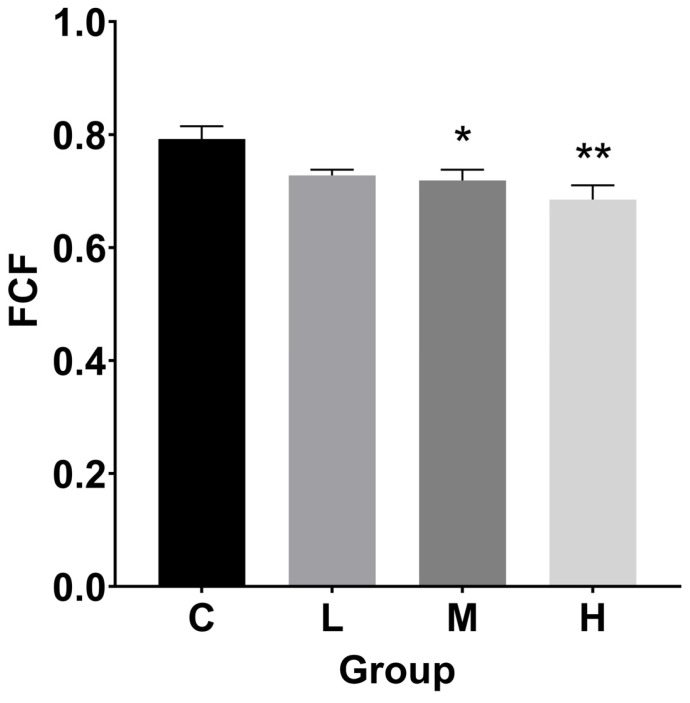
Fulton’s condition factor (FCF) for TBTC-treated haarder (n = 9). A statistically significant difference from the control is shown by an asterisk (* at *p* < 0.05 and ** at *p* < 0.01).

Compared to the control group, the FCF levels in the M and H groups were lower (Figure 3). In particular, FCF dropped significantly by 9.3% and 13.5%, respectively, in the M (*p* < 0.05) and H (*p* < 0.01) groups.

**Figure 4:**
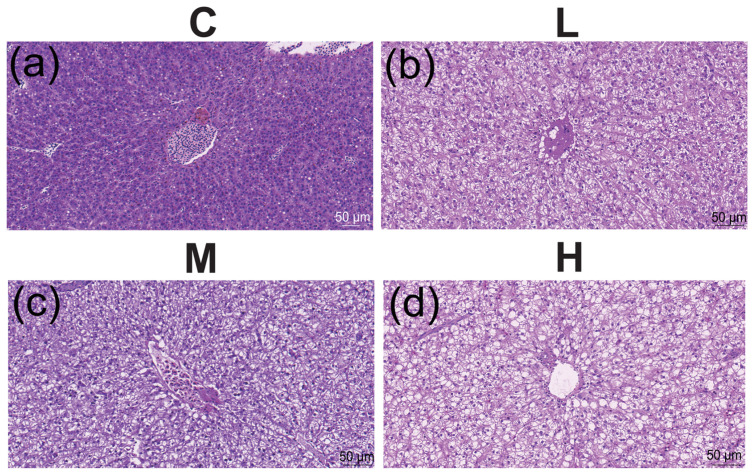
Photomicrographs of hematoxylin-eosin stained livers. (**a**) The control (C) group. (**b**) The L group. (**c**) The M group. (**d**) The H group. The sections were observed under a lens, magnified at 20×.

Using liver tissues that had been exposed to different TBTC dosages for 60 days, the histopathological alterations were identified (Figure 4a–d). In contrast to the control group’s liver, the L group’s cell gap widened, and some vacuoles started to form (Figure 4b). The M group’s liver cells had more vacuoles (Figure 4c). The nucleus moved to the cell’s periphery, and the hepatic vacuolation in the H group increased in Figure 4d.

### 3.3. ATPase Activity and Antioxidant Levels in Livers

**Figure 5:**
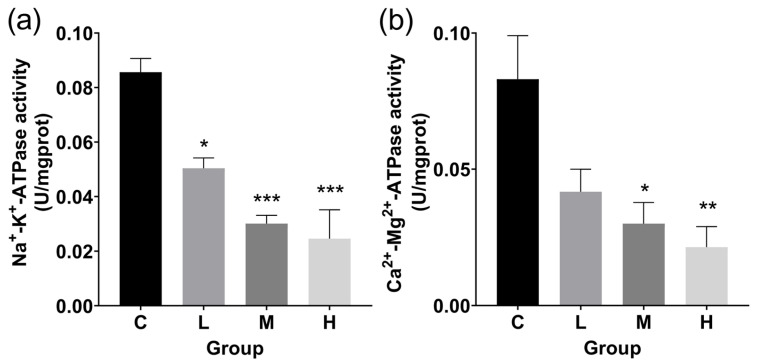
ATPase activity in the TBTC-treated groups of haarder. (**a**) Na^+^-K^+^-ATPase activity and (**b**) Ca^2+^-Mg^2+^-ATPase activity. An important variation from the control is denoted with an asterisk (* at *p* < 0.05, ** at *p* < 0.01, and *** at *p* < 0.001).

In the L, M, and H groups, TBTC significantly decreased Na^+^-K^+^-ATPase activity, which decreased by 41.2%, 64.9%, and 71.3%, respectively (Figure 5a). Ca^2+^-Mg^2+^-ATPase activity in the treatment group dramatically decreased after 60 days of exposure to TBTC, especially in the M and H groups, which saw declines of 63.9% and 74.2%, respectively (Figure 5b).

**Figure 6:**
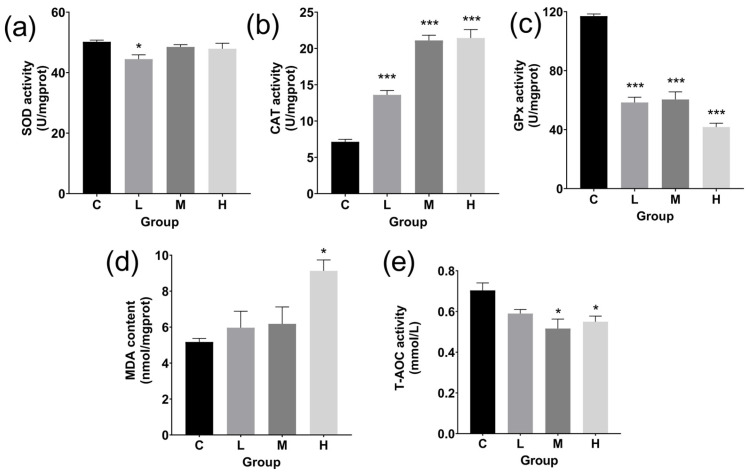
The antioxidant level and lipid peroxidation in the TBTC-treated groups of haarder. (**a**) A bar chart of superoxide dismutase (SOD) activity. (**b**) A bar chart of catalase (CAT) activity. (**c**) A bar chart of glutathione peroxidase (GPx) activity. (**d**) Malondialdehyde (MDA) content in the livers after exposure to TBTC. (**e**) Total antioxidant capacity (T-AOC) level in the liver. All samples were prepared in triplicate per group (n = 3). The asterisks indicated statistically significant differences from the control (* at *p* < 0.05 and *** at *p* < 0.001).

The L group’s SOD activity was significantly lower (*p* < 0.05) than that of the control group (Figure 6a). TBTC significantly increased CAT activity with a level of 0.001 in all treated groups (Figure 6b). The GPx activity level dropped sharply at the 0.001 level in every treatment group (Figure 6c).

MDA levels increased as the TBTC dosage increased (Figure 6d), especially in the H group, which had a level of 0.05.

Following TBTC exposure, T-AOC activity in the liver dropped by 21.9% in the H group (*p* < 0.05) and 26.6% in the M group (*p* < 0.05), as shown in Figure 6e.

### 3.4. Total Blood Cell Count and RB Detection

**Figure 7:**
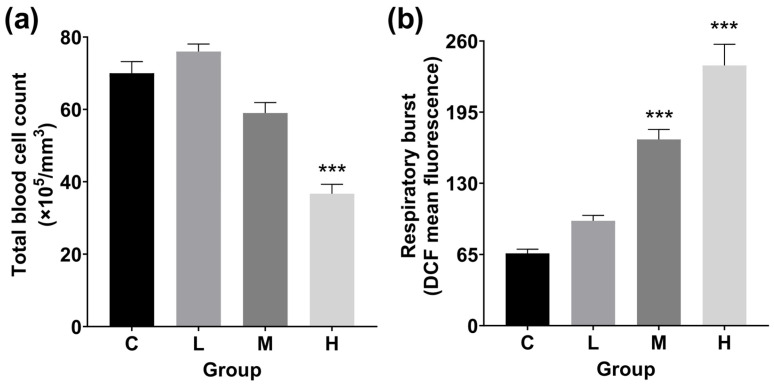
Total blood cell count and intracellular reactive oxygen species (ROS) in the TBTC-treated groups of haarder fish. (**a**) Total blood cell count after 60 d of TBTC exposure. (**b**) Respiratory burst activity in the livers. A statistically significant difference from the control was indicated by the asterisks above the column (*** at *p* < 0.001).

Due to TBTC exposure, the total blood cell count was slightly higher in the L group in Figure 7a. Total blood cell count dropped significantly in the H groups, dropping 47.6% (*p* < 0.001) from the control group.

In both the M and H groups, TBTC raised the RB level to a high level (*p* < 0.001). The amount of RB increased by 0.4, 1.6, and 2.6 times in the L, M, and H groups, respectively (Figure 7b).

### 3.5. Terminal-Deoxynucleotidyl Transferase (TdT)-Mediated dUTP Nick End Labeling (TUNEL) Detection

**Figure 8:**
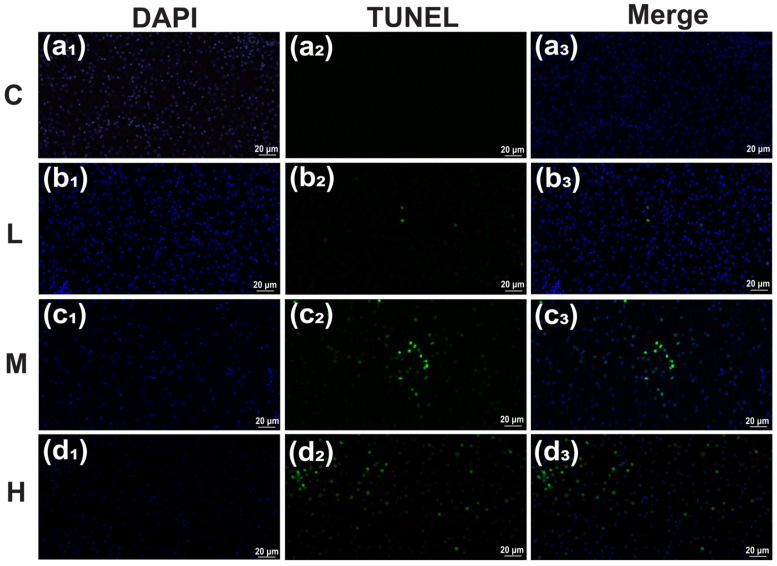
The apoptosis of liver tissue in haarder fish exposed to TBTC. (**a_1_**–**a_3_**) Control liver. TBTC-treated livers in the L (**b_1_**–**b_3_**), M (**c_1_**–**c_3_**), and H (**d_1_**–**d_3_**) groups. The blue fluorescence showed the nuclei of all cells, while the green fluorescence indicated the nuclear regions of apoptotic cells. (**a_1_**,**b_1_**,**c_1_**,**d_1_**) showed the DAPI staining results of the C group, L group, M group and H group, respectively; (**a_2_**,**b_2_**,**c_2_d_2_**) showed the staining results of the apoptotic liver cells in the C group, L group, M group and H group, respectively; (**a_3_**,**b_3_**,**c_3_d_3_**) showed the merged nuclear regions of the cells stained with DAPI and the apoptotic cells in the C group, L group, M group and H group, respectively.

**Figure 9:**
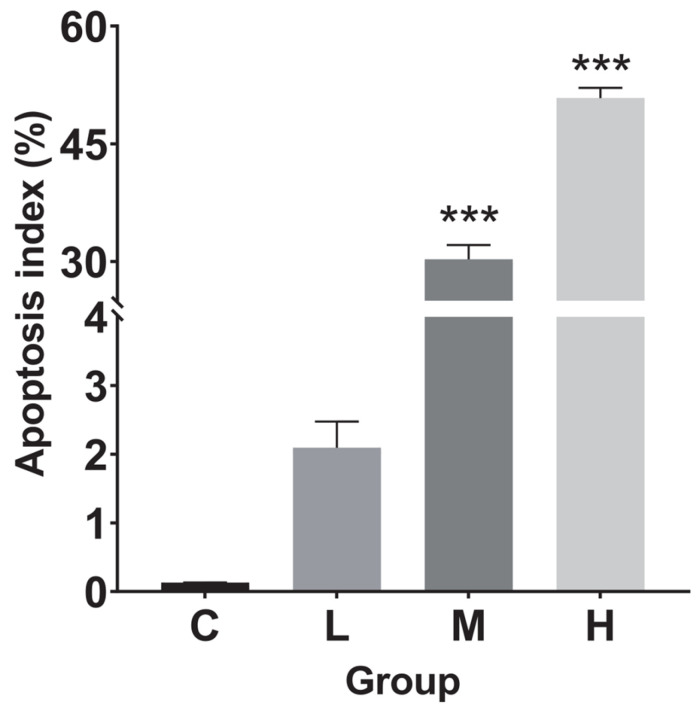
The apoptosis index of the liver tissue. The sections were observed under a lens, magnified at 40× for apoptosis livers. A statistically significant difference from the control was indicated by the asterisks above the column (*** at *p* < 0.001).

Liver tissue treated with TBTC for 60 days was examined for apoptosis using the TUNEL assay (Figure 8a–d). The apoptotic indices for the L, M, and H groups (n = 3) were 2.09 ± 0.66, 32.01 ± 3.15, and 50.71 ± 2.27%, respectively (Figure 9). In contrast to the control (0.13 ± 0.003%), the apoptotic indices of the M and H groups significantly increased, by 233.7 (*p* < 0.001) and 393.0 times (*p* < 0.001), respectively.

### 3.6. Transcriptional Levels in the Livers of TBTC-Treated Haarder

**Figure 10:**
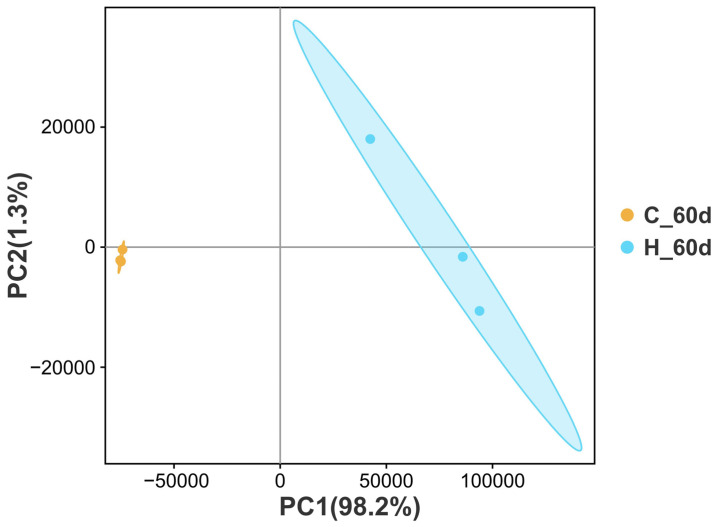
Principal component analysis (PCA) of haarder liver between the 60 d treatment of TBTC (H group, blue) and the control (C group, orange).

**Figure 11:**
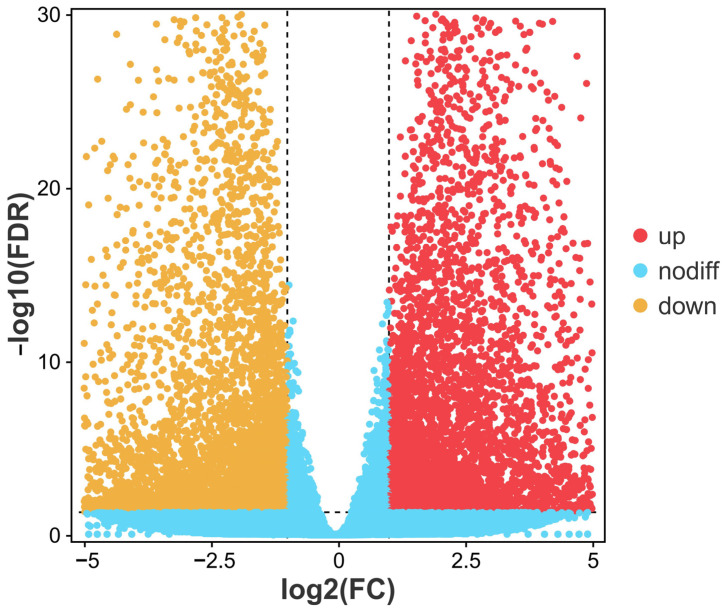
The up- and down-regulated genes in the H group depicted in a volcano plot of the differentially expressed genes (DEGs). The mean value of the log2FC is used as the ordinate (n = 3). Red dots represent up-regulated genes, while orange dots represent down-regulated genes. FDR, false discovery rate; FC, fold change.

**Figure 12:**
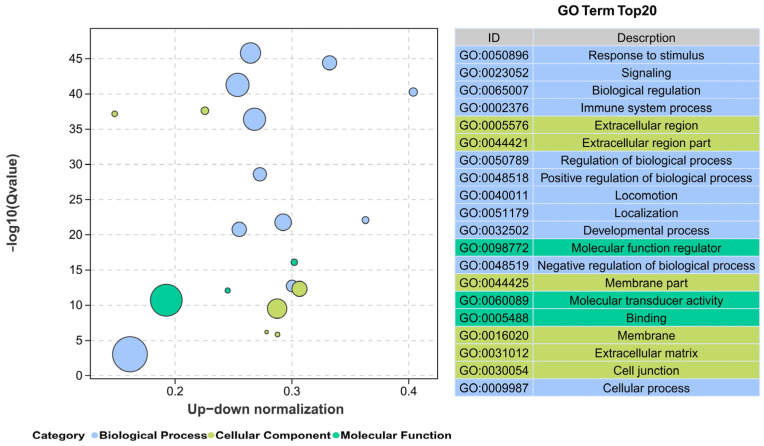
Gene Ontology (GO) enrichment analysis for haarder liver in the H group. The enrichment of the differential bubble plot was created using the significance value −log10 (Qvalue), and the top 20 GO terms are also listed on the right.

An average of 6.3 Gb clean bases per sample, or 544,443,632 clean reads, were gathered. Each sample’s Q20 and Q30 values were above 98.20% and 94.68%, respectively, indicating good transcriptome sequencing data quality (Table S1). PCA analysis of the transcriptome data showed good repeatability within each group and a significant difference between the TBTC treatment group and the C group (Figure 10). Group H had 5442 up-regulated DEGs and 5340 down-regulated DEGs in pairwise comparisons between the TBTC treatment groups and the control group (|log2FC| > 1, FDR < 0.05, Figure 11). Three categories—biological process (BP), cellular component (CC), and molecular function (MF)—are associated with the top 20 differential enrichment GO keywords in the H group (Figure 12). Among these, the BP category accounted for 55% of the DEGs, including “Response to stimulus” (GO: 0050896), “Signaling” (GO: 0023052), “Biological regulation” (GO: 0065007), “Immune system process” (GO: 0002376), “Regulation of biological process” (GO: 0050789), “Positive regulation of biological process” (GO: 0048518), “Developmental process” (GO: 0032502)”, etc. The category of CC included “Extracellular region” (GO: 0005576), “Extracellular region part” (GO: 0044421), “Membrane part” (GO: 0044425), etc. “Molecular function regulator” (GO: 0098772), “Molecular transducer activity” (GO: 0060089), and “Binding” (GO: 0005488) were three notable terms, belonging to the MF category. The DEGs related to oxidative stress, energy metabolism, and apoptosis were enriched in four classes (organismal systems, human diseases, environmental information processing, and cellular processes). The top-20 differential enrichment KEGG terms mainly included “Leukocyte transendothelial migration” (ko04670), “Proteoglycans in cancer” (ko05205), “Chemokine signaling pathway” (ko04062), and “JAK-STAT signaling pathway” (ko04630), etc.

**Figure 13:**
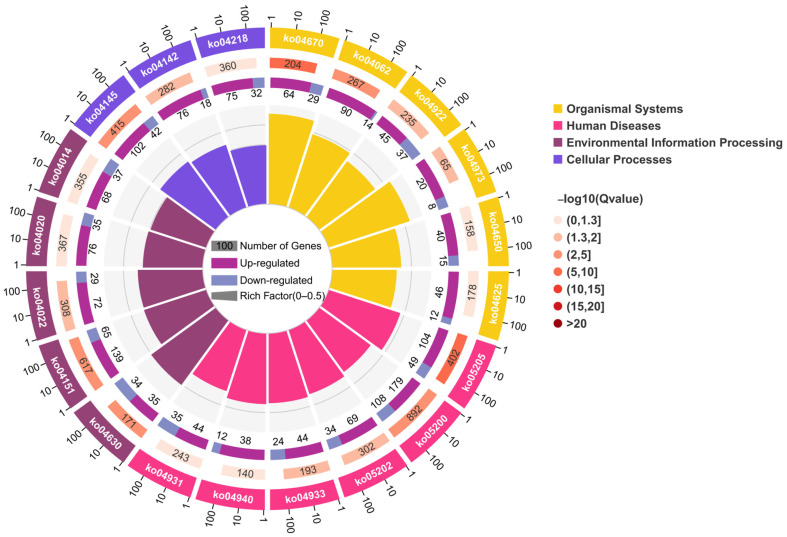
KEGG enrichment analysis for haarder liver in the H group. The circular plot of KEGG between the H group and the control group was plotted with three groups (energy metabolism dysfunction, oxidative stress, and apoptosis).

**Figure 14:**
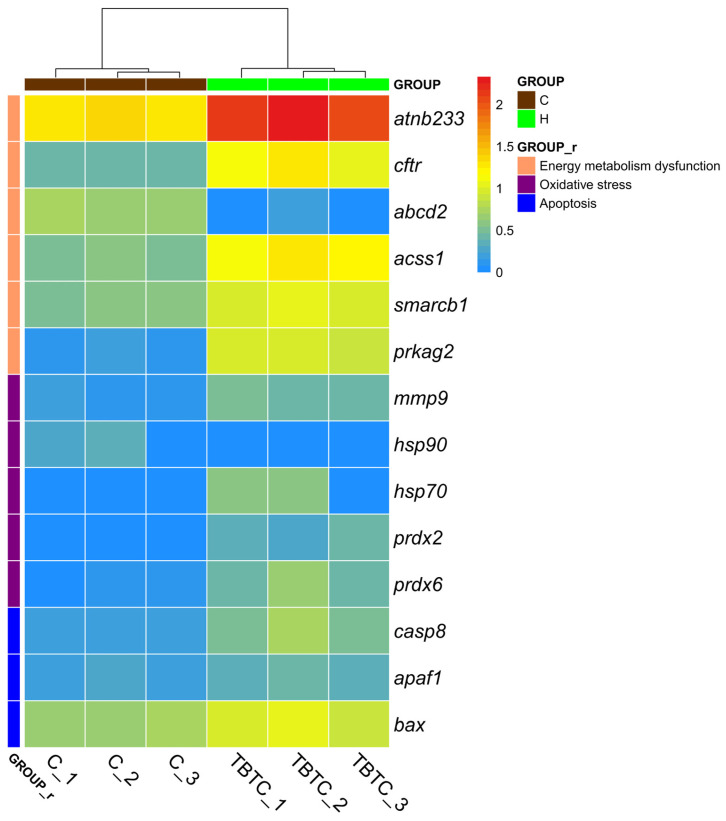
The heat map of DEGs between the H group and the control group.

Seven apoptosis-related DEGs were identified from the transcriptome data of the H group using the KEGG database (Figure 13). Of these, genes that were up-regulated were caspase 8 (*casp8*), BCL2-associated X, apoptosis regulator (*bax*), and apoptotic protease-activating factor 1 (*apaf1*) (Figure 14). In addition, TBTC in the H group also induced DEGs expression related to oxidative stress. Four up-regulated genes, including peroxiredoxin 2 (*prdx2*), peroxiredoxin 6 (*prdx6*), matrix metalloproteinase-9 (*mmp9*), and heat shock protein 70 (*hsp70*), and one down-regulated genes, such as heat shock protein 90 (*hsp90*), was identified (Figure 14). Besides, six genes associated with energy metabolism were also significantly affected by TBTC after 60 d of exposure, including three up-regulated genes, such as sodium/potassium-dependent ATPase subunit beta-233 (*atnb233*), cystic fibrosis transmembrane conductance regulator (*cftr*), and protein kinase AMP-activated non-catalytic subunit gamma 2 (*prkag2*), and three down-regulated genes, such as acyl-CoA synthettase short chain family member 1 (*acss1*), ATP-binding cassette subfamily D member 2 (*abcd2*), and SWI/SNF-related matrix-associated actin-dependent regulator of chromatin subfamily B member 1 (*smarcb1*) (Figure 14). The expression trends of these selected genes obtained from the qRT-PCR experiment in two representative differential enrichment pathways in the H group are consistent with their corresponding transcriptomic results (Figure S2). The related information of primers was listed in Table S2.

## 4. Discussion

### 4.1. The Effect of TBTC on Haarder Growth and Energy Metabolism

The process by which a chemical substance enters an organism through all exposure pathways, just as it does in the environment, is known as bioaccumulation [44]. As one of the most toxic persistent organic pollutants, the bioaccumulation of TBTC in aquatic animals may cause great ecological risks [45]. TBTC has been proven to bioaccumulate in many aquatic animals, like gastropods [46], zebrafish [47], seahorses [48], and the accumulation rate is very high. In our study, TBTC in all treatment groups had a high accumulation in the livers, and its trend became more and more obvious with the increase of concentration. This may be due to the fact that the liver, as the most important organ of fat accumulation, will lead to a large number of fat-soluble TBTC combined with liver cells [49]. This phenomenon will cause TBTC to accumulate rapidly in haarder’s body and not be discharged easily, which will have a series of effects on the whole fish body.

Many pollutants can slow down the growth of fish [50]. Two widely used growth indices, HSI and FCF, are used to assess fish growth when adverse circumstances are present [51]. In zebrafish, HSI was found to rise in female individuals, while FCF decreased when exposed to 10 and 50 ng Sn/L TBT [52]. Our study showed a similar trend in haarder after exposure to TBTC for 60 d, and showed a dose-dependent manner. The above phenomenon may be attributed to the higher bioaccumulation of TBTC in the haarder body. In addition, ATPase related to energy indirectly affected the growth and development of fish [53]. ATPase activity was found to be inhibited in TBT exposed common carp and Japanese medaka [54,55]. In this study, the enzyme activities of Na^+^-K^+^-ATPase and Ca^2+^-Mg^2+^-ATPase decreased in the treatment groups, which indicated that TBTC could inhibit their activities at the μg/L level. This indicates that the chronic exposure to TBTC may have led to haarder’s growth and development based on energy metabolism.

ATNB233 participates in osmoregulation as a subunit of sodium/potassium-dependent ATPase [56]. Additionally, PRKAG2 is the γ2 regulatory subunit of 5′ AMP-activated protein kinase, which plays a significant role in the regulation of cellular ATP metabolism [57]. The expressions of *atnb233* and *prkag2* were both up-regulated after TBTC exposure in haarder might be due to their counterbalances of ATP depletion. Many organs’ salt, fluid, and pH balance are controlled by the anion channel CFTR, which is also implicated in hypo-osmoregulation in teleosts that are acclimated to seawater. In the gills of Japanese medaka, cortisol elevated the mRNA expression of *cftr* [58]. The TBTC’s role in the hypotonic regulation of haarder may account for its increased expression in livers treated with TBTC. During the remediation of pollutants, certain ATP-dependent molecules may sustain harm [59,60]. ACSS1 is essential for the metabolism of acetate, which produces energy [61]. The SWI/SNF ATP-dependent chromatin remodelling complex includes the *smarcb1* gene as a core subunit. Each subunit contains a single ATPase, which is involved in both activating and repressing gene transcription [62]. Following TBTC exposure in haarders, the transcriptional levels of *smarcb1* and *acss1* were down-regulated along with *abcd2*, suggesting that their structures were altered to decrease expression levels.

### 4.2. Antioxidant Response of Haarder to TBTC

The antioxidant enzyme system serves as a barrier against the entry of dangerous compounds and is employed as a biomarker to indicate the level of toxicity of those substances [63]. CAT and GPx catalyze the reduction of H_2_O_2_, while SOD and other common antioxidant enzymes cleanse cells by turning toxic superoxide anion into H_2_O_2_ [64,65,66]. According to our findings, the livers’ activity of three enzymes (SOD, CAT, and GPx) declined as the dosage of TBTC increased, suggesting that TBTC contributed to the inhibition of these three enzymes’ activity. Similarly, the activities of SOD and GPx in *Perna viridis*’s gills and hepatopancreas dramatically decreased following a 24 h exposure to TBTC [67]. Furthermore, 10 and 100 ng/L of TBT decreased SOD and CAT activities in the livers of zebrafish, which once again proved the inhibitory effect of TBTC on antioxidant enzyme activities of fish and shellfish [68]. A high level of cytotoxicity and inhibition of protective enzymes is shown by the rise in lipid peroxides like MDA in fish [69]. MDA content significantly increased in the intestine of juvenile common carp exposed to TBT at a level of 7.5 µg/L for 60 days [70]. For TBTC exposed haarder, MDA content increased at 60 d, indicating that TBTC might induce ROS-mediated lipid peroxidation. T-AOC is a comprehensive indicator for measuring the functional status of the body’s antioxidant system in response to external stimulations [71]. In previous studies, TBT could weaken T-AOC in liver tissues of juvenile grass carp [72]. Our findings indicated that T-AOC was suppressed in every group treated with TBTC, indicating that the haarder’s antioxidant system had some degree of damage following the TBTC attack. We hypothesized that the haarder could first start antioxidant and detoxifying responses to counteract the negative effects of TBTC, in addition to the histological damage produced in the haarder liver. However, other toxicologically significant processes, like metabolic disorders and tissue damage, might be brought on by TBTC when antioxidation and detoxification systems fail.

Following brief focal cerebral ischemia, oxidative stress triggers *mmp9* to mediate the blood-brain barrier’s collapse [73]. *Mmp9* expression was up-regulated significantly in the 10 and 100 ng/L of TBT groups in zebrafish [47]. Our result of *mmp9* expression levels in TBTC-exposed haarder livers was found to be elevated, which implied that TBTC initiated *mmp9*-mediated oxidative stress in haarder livers. *Prdx2* is an endogenous peroxidase and has been found to reduce the oxidative burden in cells [74]. As members involved in redox processes, *prdx2* and *cat* are highly expressed compared to normal biliary epithelium cell line [75]. This phenomenon was consistent with the increases of CAT activity and *prdx2* mRNA level detected in haarder exposed to TBTC. *Prdx6*, an antioxidant, was deemed to attenuate oxidative stress on human Trabecular Meshwork cells [76]. *Prdx6* expression in haarder was up-regulated, which suggested that TBTC might reduce the oxidative stress of the liver by activating PRDX6. As an important member of heat shock proteins, the inhibition of HSP90 induced oxidative stress. The transcription levels of *hsp90* in haarder decreased after TBTC stress, which might explain that TBTC inhibited *hsp90*, which disturbed H_2_O_2_ balance and led to oxidative stress. As another member of heat shock proteins, the induction of HSP70 appeared to be a physiological response to oxidative stress of exercise [77]. The increased mRNA level of *hsp70* in haarder after TBTC exposure might explain the excessive accumulation of *hsp70* caused by oxidative stress.

### 4.3. Apoptosis Reaction of Haarder to TBTC

Apoptosis is recognized as an important toxicological feature regulated by many factors. CASP8 is an aspartate-specific cysteine protease that acts as an initiator caspase in extrinsic cell death pathways induced by tumor necrosis factor family members. The elevated expression of *casp8* in TBTC-treated haarder might indicate that the signal triggered by TBTC stimulated the expression of *casp8*, and then transmitted apoptosis signals to mitochondria [78]. *Apaf1*, as a pro-apoptotic factor, is activated by cytochrome c released from the mitochondria into the cytoplasm under the induction of the apoptosis signal. The increased mRNA level of *apaf1* in haarder after TBTC exposure confirmed that TBTC induced *apaf1* activation and promoted apoptosis in haarder. *Bax*, a member of the Bcl-2 protein family, accelerated apoptosis by adverse factors [79]. The increased mRNA level of *bax* in haarder after TBTC exposure, indicating apoptosis was exacerbated in the liver. The TUNEL results of haarder’s liver also confirmed that TBTC induced liver apoptosis.

## 5. Conclusions

The harmful effects of long-term TBTC exposure on haarders were demonstrated by this investigation. The total tin concentration in the livers increased significantly after 60 days of TBTC treatment, suggesting that TBTC had a high level of bioaccumulation in the livers and may also be the cause of a number of later bodily reactions. The histological alterations of the livers confirmed that TBTC generated severe oxidative stress and significantly impacted ATPase activity in the haarder body. Related genes at the transcriptome level demonstrated that TBTC severely harmed the haarder antioxidant enzyme system. Additionally, TBTC caused apoptosis in the liver and set off a chain reaction of functional gene responses. The distribution ranges of haarder and TBTC may overlap in some water areas, and the harmful toxicological effects of TBTC on haarder may lead to an imbalance in ecological stability [80,81]. The potential ecological hazards of TBTC in aquatic environments to fish growth and reproduction were referenced in this work.

## Data Availability

The data presented in this study are available on request from the corresponding author due to the protection policy of the research institution.

## Supplementary Materials

The following supporting information can be downloaded at: https://www.mdpi.com/article/10.3390/cimb47070526/s1.

## Abbreviations

TBT: tributyltin; TBTC, tributyltin chloride; ROS, reactive oxygen species; GPx, glutathione peroxidase; CAT, catalase; SOD, superoxide dismutase; MDA, malondialdehyde; HSI, hepatosomatic index; FCF, Fulton’s condition factor; T-AOC, total antioxidant capacity; DMSO, dimethyl sulfoxide; RB, respiratory burst; DCF-DA, 2′,7′-dichloro fluorescein diacetate; TUNEL, terminal-deoxynucleotidyl transferase (TdT)-mediated dUTP nick end labeling; qRT-PCR, quantitative real-time PCR; BUSCO, Benchmarking Universal Single-Copy Orthologs; FPKM, Fragments Per Kilobase of transcript per Million mapped reads; PCA, principal component analysis; GO, Gene Ontology; KEGG, Kyoto Encyclopedia of Genes and Genomes; DEGs, differentially expressed genes; FDR, false discovery rate; BP, biological process; CC, cellular component; MF, molecular function; casp8, caspase 8; bax, BCL2 associated X, apoptosis regulator; apaf1, apoptotic protease-activating factor 1; prdx2, peroxiredoxin 2; prdx6, peroxiredoxin 6; mmp9, matrix metalloproteinase-9; hsp70, heat shock protein 70; hsp90, heat shock protein 90; atnb233, sodium/potassium-dependent ATPase subunit beta-233; cftr, cystic fibrosis transmembrane conductance regulator; prkag2, protein kinase AMP-activated non-catalytic subunit gamma 2; acss1, acyl-CoA synthettase short chain family member 1; abcd2, ATP binding cassette subfamily D member 2; smarcb1, SWI/SNF-related matrix-associated actin-dependent regulator of chromatin subfamily B member 1.
