# Supplementary material for: Toxicity Responses from Tributyltin Chloride on Haarder (Planiliza haematocheila) Livers: Oxidative Stress, Energy Metabolism Dysfunction, and Apoptosis
Source: Curr Issues Mol Biol. 2025 Jul 8;47(7):526. doi: 10.3390/cimb47070526 (PMC12293897; doi:10.3390/cimb47070526)
Supplement: Supplementary file 1 [file cimb-47-00526-s001.zip › cimb-3712110-supplementary.pdf]

Supplementary Materials for

Toxicity Responses from Tributyltin Chloride on Haarder (*Planiliza haematocheila*) Livers: Oxidative Stress, Energy Metabolism Dysfunction, and Apoptosis

Changsheng Zhao [1,2], Anning Suo [1,2], Dewen Ding [1,2] and Wencheng Song [1,2,*]

[1] Institute for the Control of Agrochemicals, Ministry of Agriculture and Rural Affairs, Beijing 100125, China; zhaochangsheng@agri.gov.cn (C.Z.); wxdanshui001@outlook.com (A.S.); sdzcs001@foxmail.com (D.D.)
[2] Key Laboratory of Pesticide Assessment, Ministry of Agriculture and Rural Affairs, Beijing 100125, China
* Correspondence: songwencheng@agri.gov.cn

Table S1 Summary of RNA-seq results.

Sample	CleanData(bp)	Q20(%)	Q30(%)	GC(%)
C_1	6247829655	6132501499 (98.15%)	5907189567 (94.55%)	3166745801 (50.69%)
C_2	5890309634	5784287206 (98.20%)	5577874728 (94.70%)	2978395771 (50.56%)
C_3	7430007571	7292354491 (98.15%)	7026814493 (94.57%)	3768606108 (50.72%)
L_1	7042879271	6917267031 (98.22%)	6673096661 (94.75%)	3619650095 (51.39%)
L_2	6688149727	6567334124 (98.19%)	6332514908 (94.68%)	3453295286 (51.63%)
L_3	7419061342	7287441826 (98.23%)	7028783787 (94.74%)	3814922534 (51.42%)
M_1	6949251460	6822570194 (98.18%)	6575516813 (94.62%)	3522218642 (50.68%)
M_2	6500459387	6388150163 (98.27%)	6166244824 (94.86%)	3292217523 (50.65%)
M_3	5811151835	5702604706 (98.13%)	5491886896 (94.51%)	2943529690 (50.65%)
H_1	6626447803	6515380833 (98.32%)	6293384657 (94.97%)	3551923657 (53.60%)
H_2	6590042655	6467156839 (98.14%)	6228690973 (94.52%)	3429358508 (52.04%)
H_3	8058875193	7915871409 (98.23%)	7633632232 (94.72%)	4331479048 (53.75%)

Table S2 Primers used for qRT-PCR in this study.

Gene	Primer name	Primer Sequence (5’-3’)	Product size (bp)
bax	bax-S	GTTTATCTGACGCTCTGCTGTGCTC	156
	bax-A	AACCCTCTTTTGCTCTTGATATGGG	
casp8	casp8-S	ATCTGAGACTCATCCCAGTGAAAGG	101
	casp8-A	CATAGTGCTCATGCGGATCTAAAGG	
apaf1	*apaf1*-S	AATGTACGGTACTTCAGGGTCACAA	419
	apaf1-A	ATCCTTACTGTCCCGAGAACAAATC	
mmp9	mmp9-S	AGGGTTATAGTCTGTTCCTTGTGGC	479
	mmp9-A	GAGAACATCCTCGAAAGCAGAGTTA	
hsp70	hsp70-S	TCTCAATCTGCTGACCTCACATCCT	248
	hsp70-A	CCTCCTGACTTTCCTCTTTTCCTTG	
hsp90	*hsp90*-S	CGACAAGTTCTACTCTGCCTTCAGC	200
	hsp90-A	CCTTGATAGACTCGCCAGTGATGTA	
cftr	cftr-S	ATACACCCCAGTCCTCGACAATAGA	127
	cftr-A	AACCCATGATGGTACAAGTTACTCCTT	
smarcb1	smarcb1-S	AGAGTTTGTCACCACTATTGCCTAC	187
	smarcb1-A	GTCTCGGATCTTCTTCTCCATTTCA	
prkag2	prkag2-S	TGACTCAGGCAAAGTTGTGGATATT	107
	prkag2-A	GTTTCAGAGCCTGCGTCACTGTAAT	
abcd2	abcd2-S	CTCATAGGAAGGGCTACCTACGCTA	392
	abcd2-A	TGTGACCTCTTTGTAGGAGGACATG	
ef1-α	ef1-α-S	TGGTCAGATCAGTGCTGGTTATGCT	137
	ef1-α-A	ACTTTAGGGATTTGGGGTTGTCTTC	

Supplementary figure captions

Fig. S1 Calculated standard curves of the total tin (^{118}Sn).

Fig. S2 Validation of RNA-seq data by qRT-PCR (n = 6). Blue and purple columns represented the data of transcriptomic analysis and qRT-PCR, respectively. Y-axis referred to the fold changes of qRT-PCR values or FPKM values in H group compared to the control group, taken logarithm of base 2.

Fig. S1.

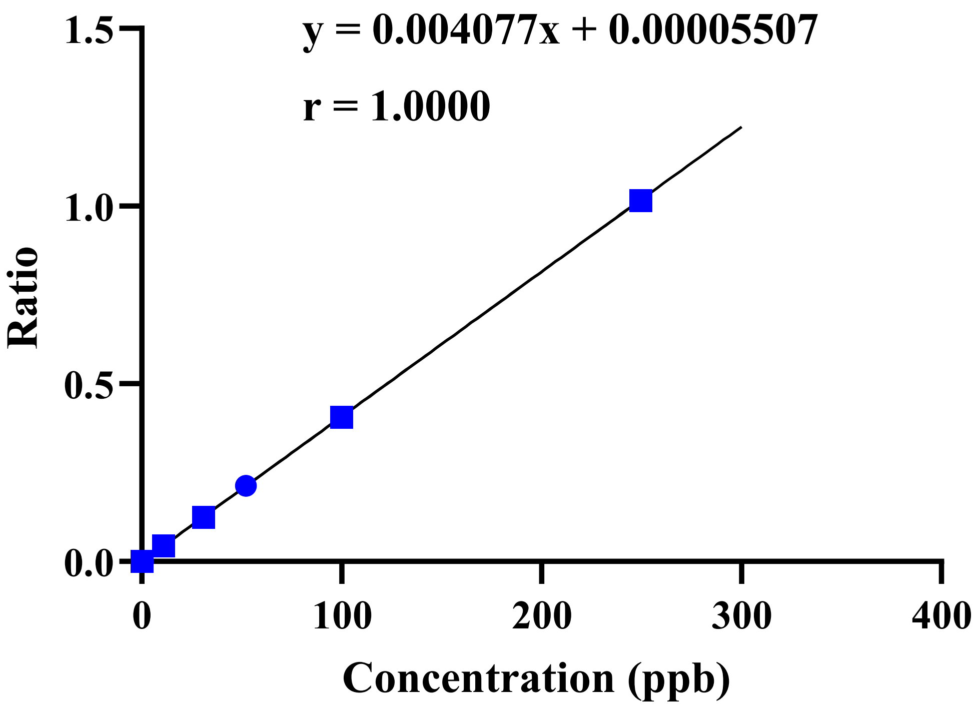
y = 0.004077x + 0.00005507
r = 1.0000
1.5
1.0
0.5
0.0
Ratio
0
100
200
300
400
Concentration (ppb)

Fig. S2.

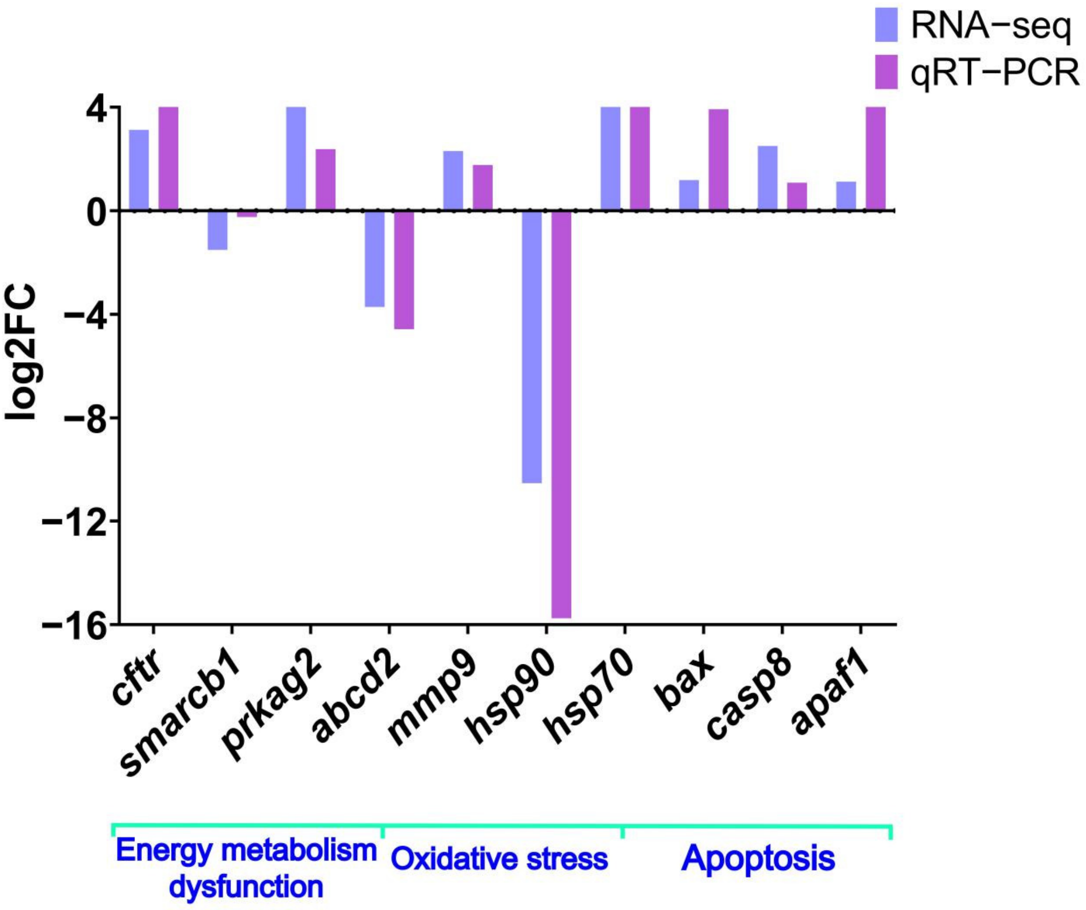
RNA−seq
qRT−PCR
log2FC
4
0
−4
−8
−12
−16
cftr
smarcb1
prkag2
abcd2
mmp9
hsp90
hsp70
bax
casp8
apaf1
Energy metabolism dysfunction
Oxidative stress
Apoptosis
